# Genetic Dissection of *qPCG1* for a Quantitative Trait Locus for Percentage of Chalky Grain in Rice (*Oryza sativa* L.)

**DOI:** 10.3389/fpls.2018.01173

**Published:** 2018-08-10

**Authors:** Aike Zhu, Yingxin Zhang, Zhenhua Zhang, Beifang Wang, Pao Xue, Yongrun Cao, Yuyu Chen, Zihe Li, Qunen Liu, Shihua Cheng, Liyong Cao

**Affiliations:** ^1^Key Laboratory for Zhejiang Super Rice Research, State Key Laboratory of Rice Biology, China National Rice Research Institute, Hangzhou, China; ^2^Nanchong Academy of Agricultural Sciences, Nanchong, China; ^3^Crop Genetic Breeding Department, Henan Agricultural University, Zhengzhou, China; ^4^National Key Laboratory of Crop Genetic Improvement, Huazhong Agricultural University, Wuhan, China

**Keywords:** chalkiness, QTL, residual heterozygous, *qPCG1*, rice

## Abstract

Rice is a pivotal cereal crop that provides the staple food for more than half of the world’s population. Along with improvements in the standard of living, people not only pay attention to the grain yield but also to the grain quality. Chalkiness is one of the most important index of grain quality. In this study, *qPCG1*, a QTL for percentage of chalky grain, was mapped in an interval with a physical distance about 139 kb on chromosome 1 by residual heterozygous line (RHL) method. *qPCG1* was incomplete dominant and the additive effect plays a major role and explained 6.8–21.9% of phenotypic variance within the heterogeneous region on chromosome 1. The effect of allele from Zhonghui9308 was decreasing the percentage of chalky grains (PCG). Microscope observation results indicated that there are great differences in the shape, structure and arrangement of starch granule between the chalky part and transparent part. Analysis of starch physicochemical properties showed that the total starch content, amylose content and chain length distribution of amylopectin changed while the protein contents were not apparently affected with the changed chalkiness. *qPCG1* had little influence on main agronomic traits and it might be useful in rice breeding for it did not bring negative effect on grain yield while reducing the chalkiness.

## Introduction

Rice is a pivotal cereal crop that provides the staple food for more than half of the world’s population (Cheng et al., 2007; Zhang, 2007; Tian et al., 2009). According to USDA, rice global cultivated area was about 160 million hectares and the total yield was about 700 million tons in 2014. Along with improvements in the standard of living, the demand for superior grain quality is increasingly becoming a prior issue in many rice production areas of the world (Cheng et al., 2007; Tian et al., 2009; Bao, 2014). Grain quality is a complex characteristic including appearance quality, milled quality, cooking quality, and nutritional quality (Wang et al., 2007; Bao, 2014). Among these properties, consumers often pay most attention to appearance (Tan et al., 2000; Wang et al., 2007; Guo et al., 2011). Grain appearance is directly related to two properties: grain shape and chalkiness (Guo et al., 2011). Chalkiness, including white core, white belly, and white back, refers to the opaque part of rice endosperm caused by the loose starch granules (Guo et al., 2011; Peng et al., 2014). Chalkiness, as a major index of the appearance quality of rice grain, is an undesirable character for it not only affects grain appearance but also has influence on physical and chemical properties, milled quality, and cooking quality (Peng et al., 2014). High chalkiness always results in more broken polished rice and worse taste so that grains with little chalkiness are more welcome for breeders and consumers.

In general, different rice cultivars show different chalkiness which means that chalkiness is mainly controlled by genetic factors. Researchers did a lot of works in detection and analysis about chalky QTLs and achieved plenty of results (He et al., 1999; Hao et al., 2009; Qin et al., 2009; Liu et al., 2011; Lu et al., 2012; Bian et al., 2013, 2014; Chen et al., 2016; Qiu et al., 2017). Until now, hundreds of QTLs of chalkiness have been detected on every chromosome in rice. For the genetic complexity and instability in chalky character, only a few QTLs about chalkiness were fine mapped or cloned up to now. *Chalk5*, as a positive regulation factor specially expressing in endosperm, which was located on chromosome 5 encoding a vacuolar H^++^-translocating pyrophosphatase, is the firstly cloned major QTL about chalkiness. It has influence on endometrial transport system by affecting the pH and further influences the subcellular ultrastructure of endosperm and formation of chalkiness (Li et al., 2014). In addition, several chalky QTLs have been fine mapped such as *qPGWC-7* (Zhou et al., 2009), *qPGWC-8* (Guo et al., 2011), and *qACE-9* (Gao et al., 2016), which is helpful for gene clone and functional analysis.

In this study, a QTL for percentage of chalky grains (PCG), *qPCG1*, was located in a 139 kb region on long arm of chromosome 1 using segregating populations developed from residual heterozygotes of a cross Xieqingzao B (XB)/Zhonghui 9308 (9308). The allele from 9308 decreased the PCG but had little influence on main agronomic traits, which could use to improve grain appearance quality without yield penalty.

## Materials and Methods

### Plant Materials

**FIGURE 1:**
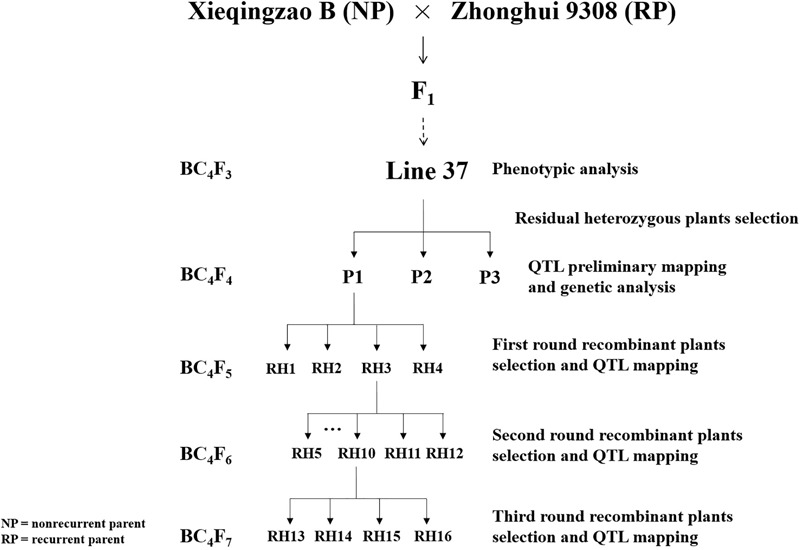
Schematic diagram of populations development in experiments.

In this study, three residual heterozygous plants in BC_4_F_4_ selected from line 37 (BC_4_F_3_) which was a residual heterozygous line (RHL) with homozygous background and a heterozygous interval on long arm of chromosome 1 derived from a backcross between non-recurrent parent XB and recurrent parent Zhonghui 9308 (9308) and their progeny populations were used for experiments (**Figure 1**). XB, an *indica* maintainer line with higher chalkiness, is one of most widely used female parent in hybrid rice breeding in China. 9308 is an *indica-japonica* hybrid restorer line with lower chalkiness. Xieyou9308, the combination of XB and 9308, was the earliest cultivar of *indica-japonica* hybrid rice with high yield and quality extensively planted in China. The populations were grown in Lingshui (LS), Hainan province and Fuyang (FY), Zhejiang province, China. All plants were grown with single plant per hole and management followed commercial rice production practices.

### DNA Extraction, PCR Protocol, and Development of Molecular Markers

The DNA of individuals was extracted from fresh leaf tissue using a modified CTAB method (Murray and Thompson, 1980). The PCR was performed using 2×Taq PCR Mix from TsingKe technology company (Hangzhou, China), PCR programs include predenaturation at 94°C for 5 min and then 30 cycles of denaturation at 94°C for 30 s, annealing at 55°C for 30 s, extension at 72°C for 30 s and a final extension of 72°C for 5 min. The PCR products were separated on 8% non-denaturing polyacrylamide gels and visualized by silver staining (Liang et al., 2014). The molecular markers for mapping were selected from the Gramene database^1^ or designed according to the resequencing data of XB and 9308 by software Oligo7.0 (**Supplementary Table 1**).

### QTL Analysis and Mapping

The genetic linkage map in this study was obtained by certain revises on the basis of the map in Shen et al. (2008) study. QTL analysis was performed with interval mapping (IM) (Lander and Botstein, 1989) in Windows QTL Cartographer Version 2.5. If *LOD* is greater than or equal to a threshold 2.0, we deduce that there is a QTL, otherwise, no QTL exists. Exact data of *LOD*, additive effect (*A*), dominant effect (*D*), and contribution to the phenotypic variance (*R*^2^) of the putative QTL were obtained from Windows QTL Cartographer Version 2.5.

Residual heterozygous method (Yamanaka et al., 2005; Du et al., 2008) was mainly used for QTL mapping in this study. The method was described as the follow. A residual heterozygous plant was gotten firstly with the heterozygous target region containing QTLs and homozygous background. After inbreeding of this plant, one heterozygous genotype plants named type 3 and two kinds of homozygous genotype plants, respectively, named NIL-type 1 which carried the allele from XB and NIL-type 2 which carried the allele from 9308 in target region were obtained. According to the phenotypic comparison between two NILs, it was deduced that there was a QTL in the target region if the phenotypic difference was extremely significant by statistical test. Then recombinant plants in this region were selected and each plant as a new residual heterozygous plant was inbred for getting new NILs. According to the difference of recombinant site and the results of phenotypic comparison between each pair of new NILs, respectively, the region would be validated and narrowed. Repeat the procedures described above until the QTL was limited in a desired interval. The schematic diagram was shown in **Supplementary Figure 1**. This method excluded the interference from background immensely and the phenotypic difference mainly come from target region as same as NILs and CSSLs. Besides the segregation populations are got by residual heterozygous plant selfing instead of hybridization or backcross to obtaining the secondary F_2_ populations for NILs or CSSLs. RHs method are useful in QTL mapping especially for the minor QTLs for these advantages.

### Scanning Electron Microscopy (SEM) Analysis and Physicochemical Properties of Starch and Contents of Protein and Lipid in Rice Endosperm

Fifty dried polished rice grains with and without chalkiness were selected, respectively, as sample for observation of endosperm cross-section. The cross-section surface was coated with gold powder and observed by scanning electron microscope with 1.0 KV voltage. The experiment was conducted in institute of Agriculture and Biotechnology, Zhejiang University.

The total starch content was measured by hydrochloric acid hydrolysis-DNS method. Amylose content was determined following GB/T 15683-2008. The chain length distribution of amylopectin was determined as described by Wang K. et al. (2015). Crude protein was determined using Kjeldahl method. Content of albumin, globulin, glutelin and prolamin were determined as described by Ju et al. (2001). All parameters related to physicochemical properties included three biological replications in NIL-type 1 and NIL-type 2. Statistical analysis was conducted by EXCEL2013.

### Chalky Phenotype and Other Agronomic Traits Measurement

In this study, plants for mapping were harvested, respectively, and 300 dried grains of each plant were randomly selected for measurement of PCG by an automatic seed counting and analyzing instrument (Model SC-E, Wanshen Ltd., Hangzhou, China).

NIL-type 1 and NIL-type 2 for survey about agronomic traits were derived from a residual heterozygous with the heterozygous region including *qPCG1* and homozygous background. Thirty lines with eight individuals per line were planted in each NIL and four individuals selected randomly in each line were used for measurement about agronomic traits including heading date (HD), plant height (PH), tilling number per plant (TN), panicle length (PL), total grain number per panicle (TGN), filled grain number per panicle (FGN), seed setting ratio (SSR), thousand grains weight (TGW), grain length (GL), grain width (GW), and the ratio of grain length to width (LWR). The mean of four individuals was as the phenotypic value of each line and the data analysis was conducted by EXCEL2013.

## Results

In this study, we reported a QTL for PCG which was named *qPCG1*. We located *qPCG1* in a 139 kb interval on long arm of chromosome 1 and analysis its genetic effect in 9308 background. Besides we further investigate the agronomic traits and conducted microscope observation and determination of physicochemical properties of the starch. The results were shown as follows.

### QTL Preliminary Detection and Development of Populations

**FIGURE 2:**
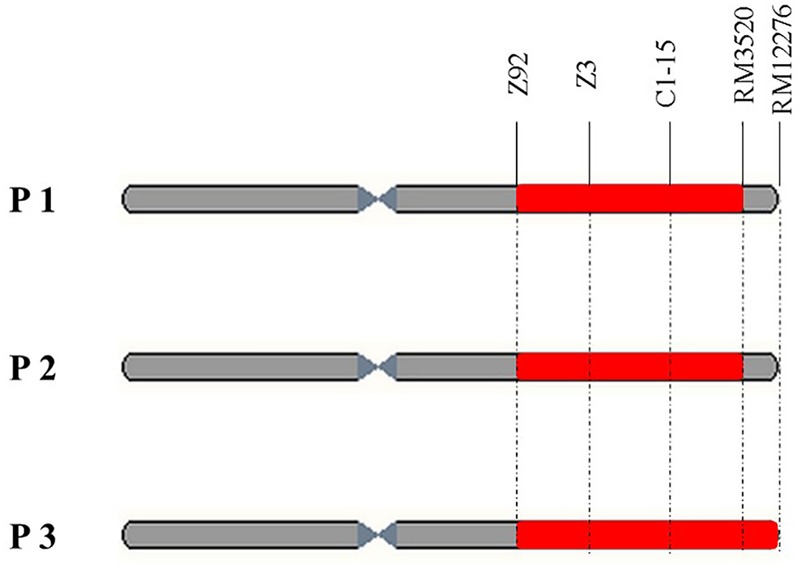
Physical maps of three residual heterozygous plants named P1, P2, and P3 (red part means heterogeneous interval and gray part means homogeneous interval).

Compared with other RHLs derived from the backcross between XB and 9308, there was no apparent difference in PH, HD, PL and grain shape among plants of line 37. By the preliminary observation in the field, we found apparent segregation in chalkiness among plants of line 37. Thus 17 plants were harvested in line 37 for survey of PCG. The results showed that PCG in the individuals of NIL-type 1 was significantly higher than those individuals of NIL-type 2 (**Table 1**). According to the results, we speculated that there might be a QTL controlling PCG in the region nearby DNA marker C1-15 on the long arm of chromosome 1. To validate the existence of QTL controlling chalkiness, Nos.2, 16, and 4, three residual heterozygous plants named P1, P2, and P3, respectively, were selected in line 37 to develop populations (**Figure 2**).

**Table 1:** Percentage of chalky grains (PCG) of 17 plants and phenotypic comparison among three genotypes in line 37.

No. plant	PCG (%)	Genotype (marker:C1–15)	Mean (%)
11	61.9	Type 1	72.8(A)
6	61.9	Type 1	
7	73.3	Type 1	
3	74.6	Type 1	
12	75.7	Type 1	
14	81.1	Type 1	
13	81.1	Type 1	
15	50.0	Type 2	57.2(B)
5	54.2	Type 2	
1	59.5	Type 2	
17	65.0	Type 2	
2	55.6	Type 3	61.5(B)
16	55.6	Type 3	
4	61.7	Type 3	
18	64.7	Type 3	
10	64.7	Type 3	
8	66.7	Type 3	

*Letters in the bracket indicate the results of multiple comparison test, capitals indicate significant difference on 0.01 level.*

### QTL Analysis

The plants harvested from three populations were divided into two parts and planted in LS and FY, respectively. The PCG was ranging from 30.83 to 43.71% in LS while from 3.92 to 9.31% in FY. The mean of PCG was significantly higher in LS than FY in three populations (**Table 2**). The results indicated that chalkiness was greatly affected by environment. The coefficient of variance was greater in FY than LS, which indicated that the degree of variation was larger in FY (**Supplementary Table 2**). The frequency distribution histogram showed the consistent result that PCG in type 1 was greater than in type 2 (**Supplementary Figure 2**). Statistic test was used for comparison the phenotypic difference among three genotypes and the results were showed in **Table 3**. The results showed significant phenotypic difference of PCG among three genotypes except population 3 in LS which suggested that there was a QTL controlling PCG in this region.

**Table 2:** Difference of percentage of chalky grains (PCG) in same populations between Lingshui and Fuyang.

Population	Place	PCG (Mean ± SD)	*P*-value
P1	Lingshui	36.75 ± 10.79	1.26E-50
	Fuyang	5.59 ± 4.37	
P2	Lingshui	36.23 ± 11.54	8.79E-49
	Fuyang	6.78 ± 4.51	
P3	Lingshui	38.83 ± 12.73	5.34E-53
	Fuyang	5.17 ± 4.25	

*P-value was determined by t-test. P < 0.01 indicates extremely significant.*

**Table 3:** Difference of percentage of chalky grains (PCG) in type 1, 2, and 3 of population P1, P2, and P3 in Fuyang (FY) and Lingshui (LS).

Place	Population	PCG (Mean ± SD)			*P*-value
		Type 1	Type 2	Type 3	
FY	P1	6.99 ± 4.78	3.92 ± 3.39	5.71 ± 4.33	0.007947
	P2	8.26 ± 5.59	4.71 ± 2.86	6.89 ± 4.13	0.007693
	P3	9.31 ± 8.38	4.56 ± 3.12	4.61 ± 2.82	0.003414
LS	P1	43.71 ± 9.94	30.83 ± 9.49	34.70 ± 9.76	0.000049
	P2	40.49 ± 10.30	31.44 ± 10.72	37.45 ± 11.69	0.014112
	P3	41.44 ± 13.38	37.51 ± 10.91	38.75 ± 13.92	0.452341

*P-value was determined by one-way analysis of variance. P < 0.05 and P < 0.01 indicate significant and extremely significant, respectively.*

Interval mapping (IM) was used for QTL detection by the Windows QTL Cartographer 2.5. In FY, the values of *LOD* in three populations were greater than 2 (**Table 4**), a threshold set beforehand, which indicated that a QTL controlling PCG was detected in the region. In LS, the QTL was detected in population P1 and P2. The basically consistent results in different populations and environments indicated there was a QTL controlling PCG on long arm of chromosome 1 which was named *qPCG1* in this region. The allele from 9308 decreased the PCG. The QTL is incomplete dominant and the additive effect plays a major role because the additive effect was greater than dominant effect in all populations. The contribution to the phenotypic variance of *qPCG1* was ranging from 6.8 to 21.9% which implied that the effect of *qPCG1* was variable in different populations and environments.

**Table 4:** Genetic effect in populations P1, P2, and P3 in Fuyang (FY) and Lingshui (LS).

Place	Populations	Interval	*LOD*	*A*	*D*	*R*^2^ (%)
FY	P1	Z92-RM3520	2.03	-1.53	0.36	6.8
	P2	Z92-RM3520	2.61	-1.88	0.51	9.8
	P3	Z92-RM12276	2.57	-2.38	-2.33	13.6
LS	P1	Z92-RM3520	4.72	-6.74	-3.07	21.9
	P2	Z92-RM3520	2.20	-5.09	0.68	9.5
	P3	Z92-RM12276	0.29	-1.97	-0.72	1.2

*LOD, likelihood of odds; A, additive effect of replacing a Xieqingzao B allele by a Zhonghui9308 allele; D, dominant effect; R^2^, contribution to the phenotypic variance.*

### Fine-Mapping of *qPCG1*

**FIGURE 3:**
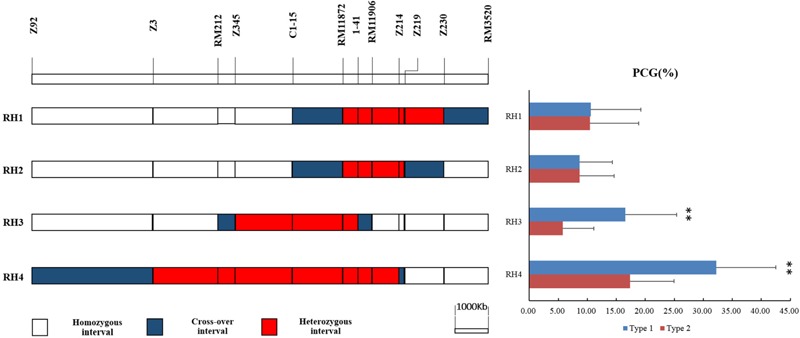
Physical maps of RH1–4 and comparison of percentage of chalky grains (PCG) between NIL-type 1 and NIL-type 2 in each population derived from RH1–4, respectively. ^∗∗^Indicates statistical significance between NILs on 0.01 level, as determined by *t*-test.

Four residual heterozygous plants in BC_4_F_5_, namely RH1, RH2, RH3, RH4, were selected from progenies of P1 and selfed to produce four sets of NILs (**Figure 3**). The PCG were compared between two genotypic lines in each set of NILs (**Figure 3**). No significant difference was detected in NILs derived from RH1 and RH2. While the PCG was significant higher in NIL-type 1 than NIL-type 2 derived from RH3 and RH4. The results indicated that *qPCG1* was located in segregating region in RH3 and RH4 but not in RH1 and 2. Therefore, *qPCG1* was mapped in the region flanked by RM212 and RM11872 with the physical distance about 3.3 Mb.

**FIGURE 4:**
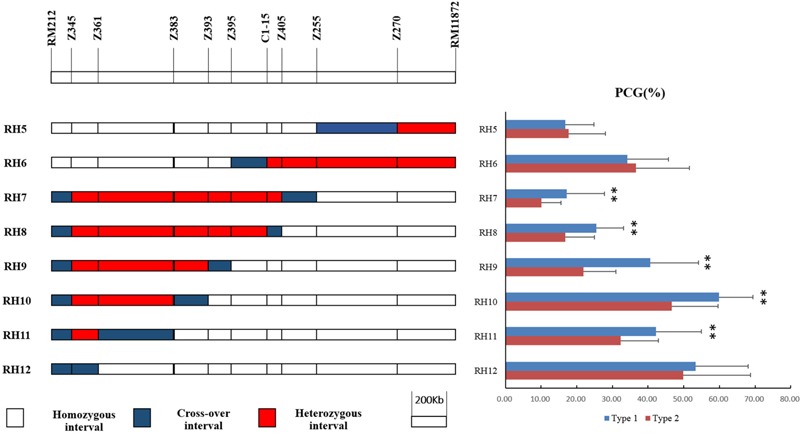
Physical maps of RH5–12 and comparison of percentage of chalky grains (PCG) between NIL-type 1 and NIL-type 2 in each population derived from RH5–12, respectively. ^∗∗^Indicates statistical significance between NILs on 0.01 level, as determined by *t*-test.

Eight recombinant plants in BC_4_F_6_, namely RH5–12, were selected from progenies of RH3 to produce eight sets of NILs (**Figure 4**). There was no significant difference in PCG between NIL-type 1 and NIL-type 2 derived from RH5, 6, and 12 while the PCG of NIL-type 1 was significant higher than that of NIL-type 2 in other populations. The results indicated that *qPCG1* did not exist in the segregating regions of RH5, 6, and 12 but in the segregating regions of RH7–11. Thus, *qPCG1* was mapped in the interval between RM212 and Z383 with the physical distance about 1,647 kb.

**FIGURE 5:**
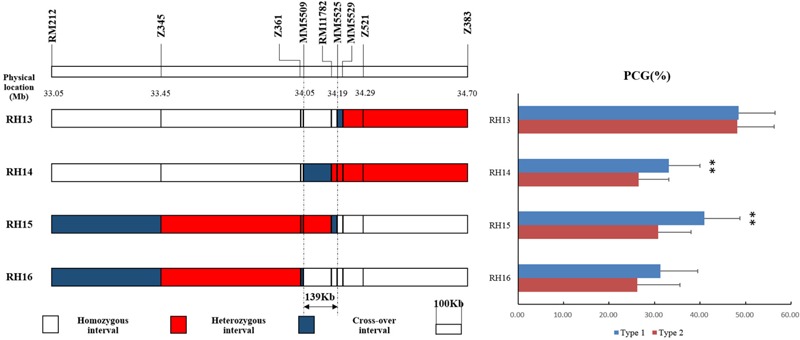
Physical maps of RH13–16 and comparison of percentage of chalky grains (PCG) between NIL-type 1 and NIL-type 2 in each population derived from RH13–16, respectively. ^∗∗^Indicates statistical significance between NILs on 0.01 level, as determined by *t*-test.

Then four recombinant plants in BC_4_F_7_ named RH13–16 were selected from progenies of RH10 to produce four sets of NILs (**Figure 5**). Significant differences were detected in the NILs derived from RH14 and RH15 but not in RH13 and RH16 (**Figure 5**), indicating that *qPCG1* was located in segregating region in RH14 and RH15 but not in RH13 and RH16. Consequently, *qPCG1* was mapped in a 139 kb region flanked by MM5509 and MM5525. The target region contains 26 predicted genes (**Supplementary Table 3**) based on Rice Genome Annotation Project Website^2^.

### Morphological Observation of Starch Structure Between Transparent Part and Chalky Part in Endosperm

**FIGURE 6:**
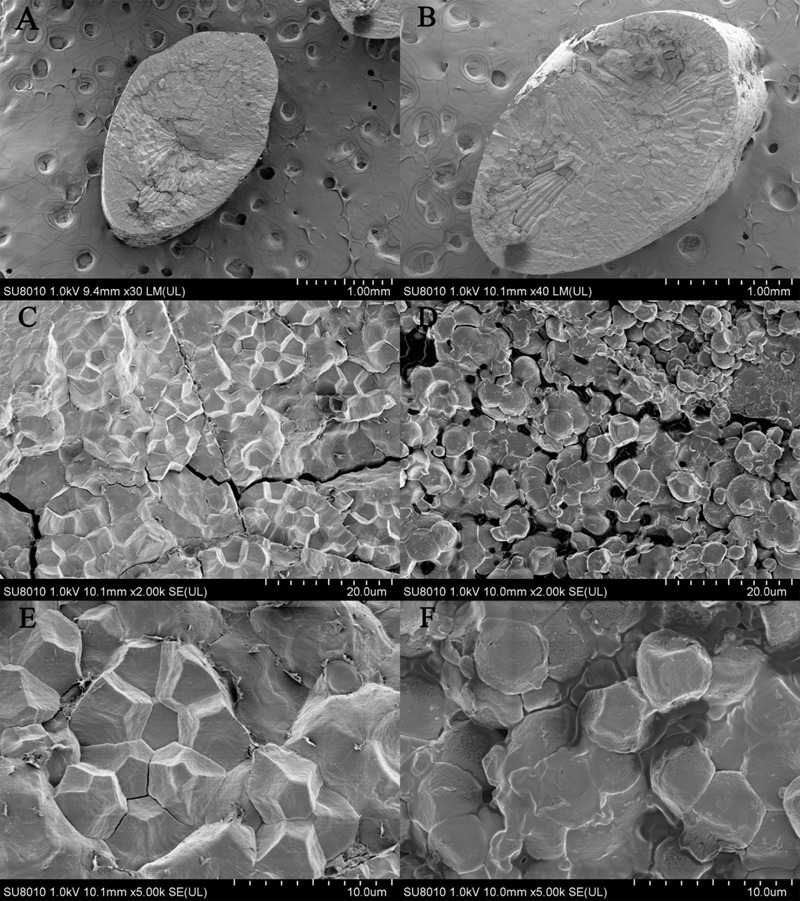
Scanning electron microscopy (SEM) analysis. **(A,C,E)** Come from transparent grain of NIL-type 2 and **(B,D,F)** come from chalky grain of NIL-type 1. The magnification is 30 times in **A**, 40 times in **B**, 2000 times in **C,D**, 5000 times in **E,F**.

Number of researches indicated that irregular morphology and incompact arrangement of starch granule in endosperm led to little light transmission consequently resulting in chalkiness generation (Zhou et al., 2009; Guo et al., 2011; Liu et al., 2012; Li et al., 2014; Wada et al., 2014; Chen et al., 2016, 2017; Gao et al., 2016; Zhang et al., 2016). In this study, scanning electron microscopy (SEM) analysis showed the consistent results (**Figure 6**). In transparent part, starch granules were regular polygon and densely packed so that there were few lacunas among the starch granules. On the contrary, starch granules in chalky part were irregular shape and loosely packed and there were lacunas among the starch granules. The difference in morphology and arrangement of starch granule in endosperm resulted in chalkiness generation.

### Difference of Physicochemical Properties of the Starch and Contents of Protein and Lipid in Endosperm Between NIL-Type 1 and NIL-Type 2

**FIGURE 7:**
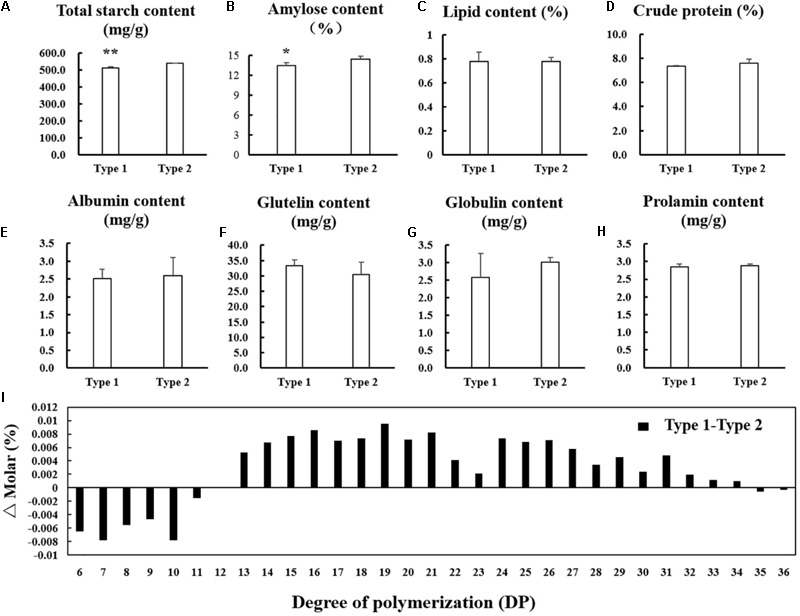
Comparison in physicochemical properties of the starch and contents of protein and lipid. **(A)** Total starch content. **(B)** Amylose content. **(C)** Lipid content. **(D)** Crude protein content. **(E)** Albumin content. **(F)** Glutelin content. **(G)** Globulin content. **(H)** Prolamin content. **(I)** Differences in the amylopectin chain length distributions. ^∗, ∗∗^ indicates statistical significance between NILs on 0.05 and 0.01 level, respectively, as determined by *t*-test.

For the significant difference in morphology and arrangement of starch granule in endosperm between NIL-type 1 and NIL-type 2, the starch physicochemical properties were also examined. The total starch and amylose contents significantly decreased in the endosperm of NIL-type 1 compared with NIL-type 2 (**Figures 7A,B**). The contents of lipid and crude protein did not changed apparently between NIL-type 1 and NIL-type 2 (**Figures 7C,D**) and no significant difference occurred in albumin, glutelin, globulin and prolamin between NIL-type 1 and NIL-type 2 (**Figures 7E–H**). To further analyze the fine structure of amylopectin, its chain length distribution was determined. Compared to NIL-type 2, the proportion of short chains with degree of polymerization (DP) values between 6 and 12 decreased, whereas the proportion of intermediate chains with DP values between 13 and 24 and the proportion of long chains with DP values between 25 and 33 increased in NIL-type 1 (**Figure 7I**).

### Evaluation on the Application Value of *qPCG1* in Rice Breeding

**FIGURE 8:**
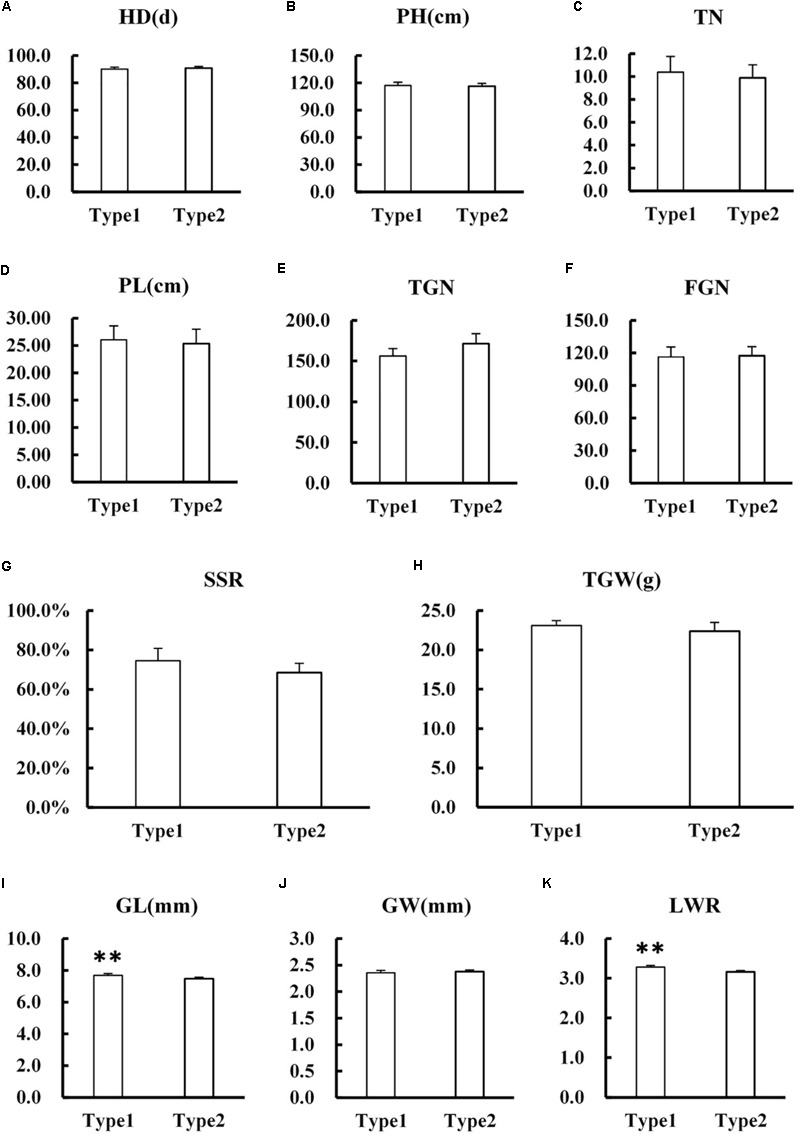
Comparison in main agronomic traits between NIL-type 1 and NIL-type 2. **(A)** Heading date (HD). **(B)** Plant height (PH). **(C)** Effective tillering number per plant (TN). **(D)** Panicle length (PL). **(E)** Total grain number per panicle (TGN). **(F)** Filled grain number per panicle (FGN). **(G)** Seed set rate (SSD). **(H)** Thousand grain weight (TGW). **(I)** Grain length (GL). **(J)** Grain width (GW). **(K)** Grain length-width ratio (LWR). ^∗∗^ indicates statistical significance between NILs on 0.01 level, as determined by *t*-test.

Several agronomic traits including HD, PH, TN, PL, TGN, FGN, SSR, TGW, GL, GW, and LWR were surveyed between NIL-type 1 and NIL-type 2. The results showed that there was no significant difference in most agronomic traits such as HD, PH, TN, PL, TGN, FGN, SSR, TGW, and GW between NIL-type 1 and NIL-type 2 (**Figure 8**). Although the difference was detected in GL which consequently resulted in the difference in LWR, the difference of GL was only 0.22 mm and did not result in significant difference in grain weight between NIL-type 1 and NIL-type 2. According to the results, we deduced that *qPCG1* had little influence on grain yield while changed the PCG which meant it might be useful for rice breeding.

## Discussion

Grain quality is a very important breeding objective in rice and chalkiness is one of most undesirable qualitative characteristic for breeders and consumers. Recently, plenty of researches about grain chalkiness have been done. Peng et al. (2014) detected 79 QTLs controlling chalkiness by five populations in two environments. Zhao et al. (2016) detected 140 QTLs controlling chalkiness by reciprocal cross between Teqing and Lemont in nine environments. Liu et al. (2013) detected 15 QTLs controlling chalkiness by MCIM, ICIM, and MIMR three methods. Li et al. (2004) detected two QTLs controlling chalkiness by an interspecific backcross population derived from cultivated Asian (*O. sativa* L.) and African (*O. glaberrima* S.) rice. Mei et al. (2013) detected 25 QTLs controlling chalkiness by three different RIL populations. Wan et al. (2005) detected 9 QTLs controlling chalkiness in eight environments. Although numbers of QTLs about chalkiness were detected, a few of them were fine mapped and cloned. *Chalk-5*, which encodes a vacuolar H^+^-translocating pyrophosphatase, is the first cloned QTL about chalkiness. *qPGWC-7* and *qPGWC-8*, two QTLs controlling percentage of grains with chalkiness (PGWC), were fine mapped on chromosome 7 and 8, respectively, by different populations. *qACE-9*, a QTL controlling area of chalky endosperm (ACE), was fine mapped on chromosome 9 and *OsAPS1* was speculated as a candidate gene. The main reasons why only a few QTLs about chalkiness were cloned are genetic complexity and environmental sensitivity. Besides genetic factors, chalkiness is also influenced by many external factors. In general, temperature has a great effect on grain chalkiness and high temperature increases chalkiness in grains (Tsukaguchi and Iida, 2015; Wang Z.M. et al., 2015; Jing et al., 2016b; Kaneko et al., 2016; Suriyasak et al., 2017). Li et al. (2011) study indicated that compared with DHT (day high temperature), NHT (night high temperature) exerted less effect on chalkiness. Elevated ozone and carbon dioxide both increased grain chalkiness (Wang et al., 2014; Jing et al., 2016b) and ozone sensitive varieties were more susceptible by ozone (Jing et al., 2016a). Zhou et al. (2015) study indicated that degree of endosperm chalkiness (DEC) and PGWC were decreased with increasing N rates in both dry and wet seasons. Low light treatment in early stage (from transplanting to spikelet differentiation) decreased chalkiness (Liu et al., 2006) but during the grain-filling stage low light treatment increased chalkiness (Ren et al., 2003). Researchers have made great progress in grain quality but chalkiness is still a serious problem for its complicacy. In the future, from mapping to clone, from functional analysis to network regulatory, more work about chalkiness should been done. The more information about genetic mechanism of chalkiness we get, the more helpful it is for improvement in grain chalkiness.

In this study, *qPCG1*, a QTL controlling PCG, was mapped in an interval with a physical distance about 139 kb on chromosome 1 by residual heterozygous method. The additive effect is from 1.53 to 6.74 and dominant effect is from 0.36 to 3.07, explained 6.8–21.9% of phenotypic variance. In previous studies, several QTLs controlling PGWC were detected on long arm of chromosome 1 (Wan et al., 2005; Chen et al., 2011; Liu et al., 2011; Bian et al., 2013; Sun et al., 2015). By the analysis of allelism, the intervals of *qPGWC-1* which was detected by Wan in four environments (Wan et al., 2005) and *qPGWC-1a* which was detected by Chen et al. (2011) in 2 years were similar to *qPCG1*. The intervals of *qPGWC-1* and *qPGWC-1a* are approximate 5.0 and 1.6 Mb, respectively, which both include the target interval of *qPCG1*. The contribution to the phenotypic variance, respectively, are from 16.3 to 18.7% and from 6.9 to 23.2%. Besides *qPGWC-1a* reported by Liu et al. (2011) was close to *qPCG1* with the physical distance about 290 kb. Although four QTLs are located in the similar interval with the similar contribution to the phenotypic variance, the intervals of four QTLs should be further narrowed enough to validate whether they are the same QTL or not.

RHs method was mainly used for QTL mapping in this study. The advantage of this method is to exclude the interference from the background as far as possible, reduce the phenotypic variation and improve reliability of genetic analysis. On the other hand, the contribution to the phenotypic variance calculated in this study do not present the one throughout whole genome but a limited range of heterogeneous region. The problem do exist not only in RHs, but in NILs, CSSLs and other populations with highly consistent background. RIL population with numbers of recombination site in the whole genome may be useful in estimating the contribution to the phenotypic variance throughout whole genome. Another problem is that the estimates of QTL effects can be biased when QTLs are linked by IM. For these reasons, the contribution of *qPCG1* might be higher in this study.

Statistical test results show difference in GL between NIL-type 1 and NIL-type 2, which consequently resulted in the difference in LWR, but the difference in GL has little influence on grain weight. After referring to relevant papers and Gramene database, we found that there was a minor QTL about TGW named *qTGW-1.2b* in the similar interval to *qPCG1* which affected not only grain weight but GL (Wang L. et al., 2015). The interval of *qTGW-1.2b* was approximate 510 kb which included about 23 kb overlapped part to the interval of *qPCG1*. Unlike *qPCG1, qTGW-1.2b* brought the difference not only in GL but in grain weight and HD. We will detect the difference of GL and other traits about grain shape between NILs in the next seasons to exclude interference of false positive. At the same time sequence comparison of genes in the 23 kb overlapped interval and further narrowing the mapping interval will be conducted to ascertain whether they are single QTL with pleiotropy or two QTLs linked tightly.

There are 26 genes in the target interval based on Rice Genome Annotation Project Website. The products of nine genes are expressed protein or hypothetical protein. Seven of them code the cytochrome P450 family proteins. The proteins in this family are functional in many aspects such as disease resistance, stress resistance, herbicide resistance, regulation in Gibberellic acid (GA) and Brassinosteroid (BR) and so on. Three of them belong to certain domain containing protein and two of them are glucosyltransferases. Both of domain containing protein and glucosyltransferase have a lot of biologic functions. The rest of genes products are 50S ribosomal protein, retrotransposon protein, thylakoid lumenal 20 kDa protein, cyclin, and proline-rich family protein, respectively. To validate which one is the candidate gene of *qPCG1*, further studies such as selection of new recombinant plants for narrowing the interval and comparison sequence of genes are in progress.

Years of studies shows that *qPCG1* expresses both in LS and FY and the effect of allele from 9308 is always decreasing the PCG. At the same time, *qPCG1* shows little influence on many agronomic traits such as HD, PH, tillering number, PL, grain number, seed set rate, and so on. All the results indicate that *qPCG1* might be useful in rice breeding. The follow work is narrowing the target interval and analysis genes in the interval to validate the candidate gene of *qPCG1*. Next, genetic effect of *qPCG1* will be further analyzed in different genetic backgrounds such as XB after validating the candidate gene for the existence of epistasis in other background might results in the change of genetic effect of *qPCG1*. Then allelism analysis will be done in main parents of rice hybridization breeding to ascertain that the allele in the locus of *qPCG1* is from XB or 9308 in each parent. All of the above are helpful to ensure the value of *qPCG1* in rice breeding.

Microscope observation results indicate that in transparent part the starch granule shape is regular polygon and there is little interspace among the starch granule. On the contrary, in chalky part the starch granule shape is irregular and the arrangement is loose. The significant difference in the shape, structure and arrangement of starch granule results in chalkiness generation. Physicochemical properties of the starch results indicate that total starch content and amylose content is lower and the proportion of short chains with DP values between 6 and 12 decreased, whereas the proportion of intermediate chains with DP values between 13 and 24 and the proportion of long chains with DP values between 25 and 33 increased in NIL-type 1 compared NIL-type 2. Some studies indicated that higher chalkiness might be accompanied with lower amylose content (Kang et al., 2005; Wang et al., 2008; Gao et al., 2016), but some other studies indicated that higher chalkiness might be accompanied with higher amylose content (Fujita et al., 2007; Guo et al., 2011) and in Zhou’s report, there was no apparent change in amylose content between higher chalkiness material and lower one (Zhou et al., 2009). Thus we speculated that chalkiness was not a simple correlation but a complicated relationship with amylose content and further studies should be conducted about the relationship between chalkiness and amylose content. Unlike the inconclusive relationship between chalkiness and amylose content, high chalkiness was accompanied with short chains decrease and long chain increase of amylopectin in this study as same as previous studies (Yamakawa et al., 2007; Lin et al., 2016; Cai et al., 2018). In Umemoto’s report, lower temperature led to an increased proportion of chains of DP 6–13 and decreased the percentage of chains with DP 20–27 and DP 44–54 and no statistically significant differences were observed in the proportions of each fraction of amylopectin between non-waxy and waxy varieties (Umemoto et al., 1999). Although no direct evidences about the relationship between chalkiness and amylopectin were given in this study, according to the results and the positive correlation between temperature and chalkiness, we conjectured that low chalkiness might be accompanied with the increase in short chain and decrease in long chain of amylopectin. These results suggest that alterations of amylopectin structure are involved in grain chalkiness. No significant difference was detected in lipid content, protein content and component between NILs in this study which might indicated that *qPCG1* had no influence on lipid and protein in endosperm.

In summary, *qPCG1* is a QTL controlling PCG mapped on long arm of chromosome 1. *qPCG1* is incomplete dominant and the additive effect plays a major role and explained 6.8–21.9% of phenotypic variance within the heterogeneous region on chromosome 1. The effect of allele from 9308 is decreasing the PCG. There are great differences in the shape, structure, and arrangement of starch granule between the chalky part and transparent part. *qPCG1* changes the physicochemical properties of starch but has no apparent influence on contents of lipid and protein in endosperm. *qPCG1* has little influence on main agronomic traits while changing the chalkiness. All the results provide valuable information for gene clone and functional analysis of *qPCG1* and utilization in rice breeding.

## Author Contributions

LC, SC, and YZ conceived and designed the study. AZ conducted all the experiments and guided the observation of chalkiness and the acquisition of genotype data with BW, PX, YoC, and ZL. AZ analyzed the data and formatted the figures with YZ, ZZ, YuC, and QL. AZ and YZ wrote the paper. LC and SC reviewed and edited the manuscript. The manuscript was read and approved by all the authors.

## Conflict of Interest Statement

The authors declare that the research was conducted in the absence of any commercial or financial relationships that could be construed as a potential conflict of interest.

## Footnotes

1 www.gramene.org

2 http://rice.plantbiology.msu.edu
