# Supplementary material for: Genetic Dissection of qPCG1 for a Quantitative Trait Locus for Percentage of Chalky Grain in Rice (Oryza sativa L.)
Source: Front Plant Sci. 2018 Aug 10;9:1173. doi: 10.3389/fpls.2018.01173 (PMC6095994; doi:10.3389/fpls.2018.01173)
Supplement: Supplementary file 1 [file Data_Sheet_1.docx]

Supplementary Material

**Genetic Dissection of *qPCG1* for a Quantitative Trait Locus for Percentage of Chalky Grain in Rice (*Oryza sativa* L.)**

**Aike Zhu^1,2†^, Yingxin Zhang^1†^, Zhenhua Zhang^1^, Beifang** **Wang^1^, Pao Xue^1^, Yongrun Cao^1,3^, Yuyu Chen^1,4^,** **Zihe Li^1^,** **Qunen Liu^1^, Shihua Cheng^1,*^****, Liyong Cao^1, *^**

*** Correspondence:** Corresponding authors: Shihua Cheng: [shcheng@mail.hz.zj.cn](mailto:shcheng@mail.hz.zj.cn)

Liyong Cao: [caolycgf@mail.hz.zj.cn](mailto:caolycgf@mail.hz.zj.cn)

**Supplementary Table 1.** Molecular markers for mapping

| Markers | Forward primer (5' - 3') | Reverse primer (5' - 3') | Physical location |
| --- | --- | --- | --- |
| Z92 | GCACGGTTCTCTTTCTGTGATAGC | TTTCGAAGACCGTAGTAGCAGTGG | 26,486,863 |
| Z3 | AAAGAAAACATCCTTGACTTCCCACC | CCTTCTGAGAGCGCCTGGGTAT | 30,093,838 |
| RM212 | CCACTTTCAGCTACTACCAG | CACCCATTTGTCTCTCATTATG | 33,054,687 |
| Z345 | TTTTGAGATTTGGACTCACG | TCTTCTTACCTACACTTAGTCCT | 33,450,419 |
| Z361 | ATAATTGTTTTCTGGGTTCGG | CTACCTCTCTGTTCCCTCAC | 34,041,678 |
| MM5509 | ATCTACAAACGGAACCGTCG | CATAGATCGCACTCGGGTCT | 34,046,636 |
| RM11782 | GGAGTGTTTGTAGGACTGACACG | ACATTACCCGAATCAGGATAGG | 34,173,600 |
| MM5525 | GGCCTTAATAGGAAGTTGTTTCC | CCCGCACAGGTGTACAATC | 34,185,183 |
| MM5529 | AAAATTGGACACGGAAACCC | CCATCCCACCATAGGAGCTA | 34,200,601 |
| Z521 | TTGAACAGGAGGTCTAGTGA | CGCTCTTCCTCCATTAGACA | 34,285,570 |
| Z383 | GTTGCACGCAAAACGCCGCTA | GTCTTGCTTGCTCTCGGTGG | 34,702,058 |
| Z393 | ATGTTTCAATTATTGCGGCTG | TATTATCAAAATTTGGTAAGGC | 34,895,116 |
| Z395 | TTAACACCTATATCAAACGGAG | GCCGCCGCCAACTCAGGAA | 34,994,733 |
| C1-15 | CAACCCCTCCAAATACCTGA | ACCGTGTTCATGCCTTTCAC | 35,191,728 |
| Z405 | ACGCTCTATTTGTTTTCGCTT | GCCTGTTTGTTTCTCATCGTAT | 35,263,542 |
| Z255 | GAAACATTGTAAATACACTAGG | AATCCGAAAACACTTACCCTC | 35,430,824 |
| Z270 | AAAGATGAAGTTGGGAACTCAC | TAATGGACACTTTATTTTCCGT | 35,717,717 |
| RM11872 | CATCCCAATCTGGCCTTGACTAAGC | TTCTCGGGAGGTGGAGGACAGG | 36,389,840 |
| 1-41 | GAGGTACTTCCTCCGTTTCAC | AGTCAGCTCACTGTGCAGTG | 36,735,315 |
| RM11906 | GCTCGCTAAACACACTAGACATTTCC | CATAGGCAGATGAAGGCAACTCG | 37,123,128 |
| Z214 | CGCGCAAGTGACCTCGAT | GCACAATAGGCTAACTATACCTCT | 38,044,545 |
| Z219 | GGTGTTTCCTCGTCTGTT | TAAGTATAAGCATAACAAAACGAC | 38,240,460 |
| Z230 | CTGGAACAACTTTCGATGCTG | CATGAGAATGCCTGACTGGAT | 38,803,308 |
| RM3520 | CTGCAAATGCACAGGAATCAGG | TCCTCTCGCCTTTCTTTCTCTCC | 40,253,719 |
| RM12276 | GTCGACGGCTTCCTCAAGATTGG | TGAGACCTCTGTGAAGGCACTCG | 43,044,320 |

**Supplementary Table 2.** Phenotypic value of percentage of chalky grains (PCG) and descriptive statistic in type 1, 2 and 3 of P1, P2 and P3 in Fuyang (FY) and Lingshui (LS)

| Place | Population | Genotype | PCG(%) | SD | CV | Range | Skewness | Kurtosis |
| --- | --- | --- | --- | --- | --- | --- | --- | --- |
| FY | P1 | Type 1 | 6.99 | 4.78 | 0.684 | 0.50-20.22 | 1.021 | 0.524 |
|  |  | Type 2 | 3.92 | 3.39 | 0.865 | 0.17-13.50 | 1.326 | 1.322 |
|  |  | Type 3 | 5.71 | 4.33 | 0.759 | 0.60-23.33 | 1.679 | 4.322 |
|  | P2 | Type 1 | 8.26 | 5.59 | 0.676 | 1.50-27.90 | 1.416 | 3.022 |
|  |  | Type 2 | 4.71 | 2.86 | 0.607 | 0.33-9.83 | 0.498 | -0.858 |
|  |  | Type 3 | 6.89 | 4.13 | 0.599 | 0.67-17.17 | 0.391 | -0.729 |
|  | P3 | Type 1 | 9.31 | 8.38 | 0.900 | 3.17-31.83 | 2.578 | 7.205 |
|  |  | Type 2 | 4.56 | 3.12 | 0.684 | 0.17-11.67 | 0.702 | -0.308 |
|  |  | Type 3 | 4.61 | 2.82 | 0.611 | 0.67-13.50 | 1.237 | 1.691 |
| LS | P1 | Type 1 | 43.71 | 9.94 | 0.227 | 14.40-62.54 | -0.752 | 1.654 |
|  |  | Type 2 | 30.83 | 9.49 | 0.308 | 17.24-56.23 | 1.193 | 2.265 |
|  |  | Type 3 | 34.70 | 9.76 | 0.281 | 10.21-51.71 | -0.538 | 0.300 |
|  | P2 | Type 1 | 40.49 | 10.30 | 0.254 | 27.00-63.38 | 0.518 | -0.339 |
|  |  | Type 2 | 31.44 | 10.72 | 0.341 | 5.96-52.07 | 0.219 | -0.011 |
|  |  | Type 3 | 37.45 | 11.69 | 0.312 | 12.50-65.41 | 0.631 | 0.139 |
|  | P3 | Type 1 | 41.44 | 13.38 | 0.323 | 16.56-63.48 | 0.099 | -0.801 |
|  |  | Type 2 | 37.51 | 10.91 | 0.291 | 12.52-54.16 | -0.326 | -0.752 |
|  |  | Type 3 | 38.75 | 13.92 | 0.359 | 17.45-66.35 | 0.285 | -1.096 |

**Supplementary Table 3.** Number and description of genes in the region between marker MM5509 and MM5525 on the long arm of chromosome 1

| Genes’ Number | Description |
| --- | --- |
| 7 | cytochrome P450 |
| 6 | expressed protein |
| 3 | hypothetical protein |
| 1 | retrotransposon protein |
| 1 | 50S ribosomal protein |
| 1 | thylakoid lumenal 20 kDa protein |
| 1 | cytokinin-N-glucosyltransferase 1 |
| 1 | indole-3-acetate beta-glucosyltransferase |
| 1 | cyclin |
| 1 | proline-rich family protein |
| 1 | tubulin/FtsZ domain containing protein |
| 1 | UBA/TS-N domain containing protein |
| 1 | OsFBX27 - F-box domain containing protein |

**Supplementary Figure 1.** **Schematic diagram of RH method**

**
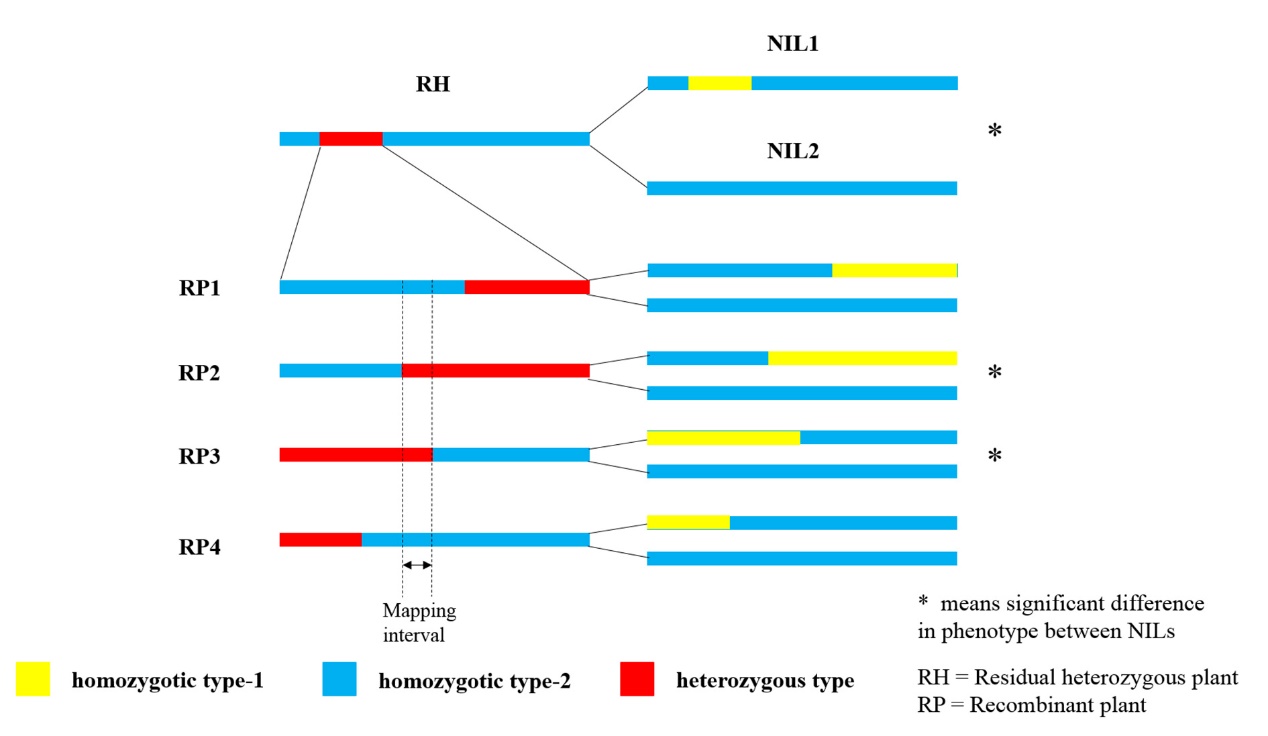
**

**Supplementary Figure 2.** Frequency distributions of type 1, type 2 and type 3 in three populations in Lingshui and Fuyang. (A), (B) and (C) are frequency distributions of populaiton1, 2 and 3 respectively in Lingshui and (D), (E) and (F) are frequency distributions of populaiton1, 2 and 3 respectively in Fuyang. Blue, red and green represent type 1, 2 and 3 respectively.**
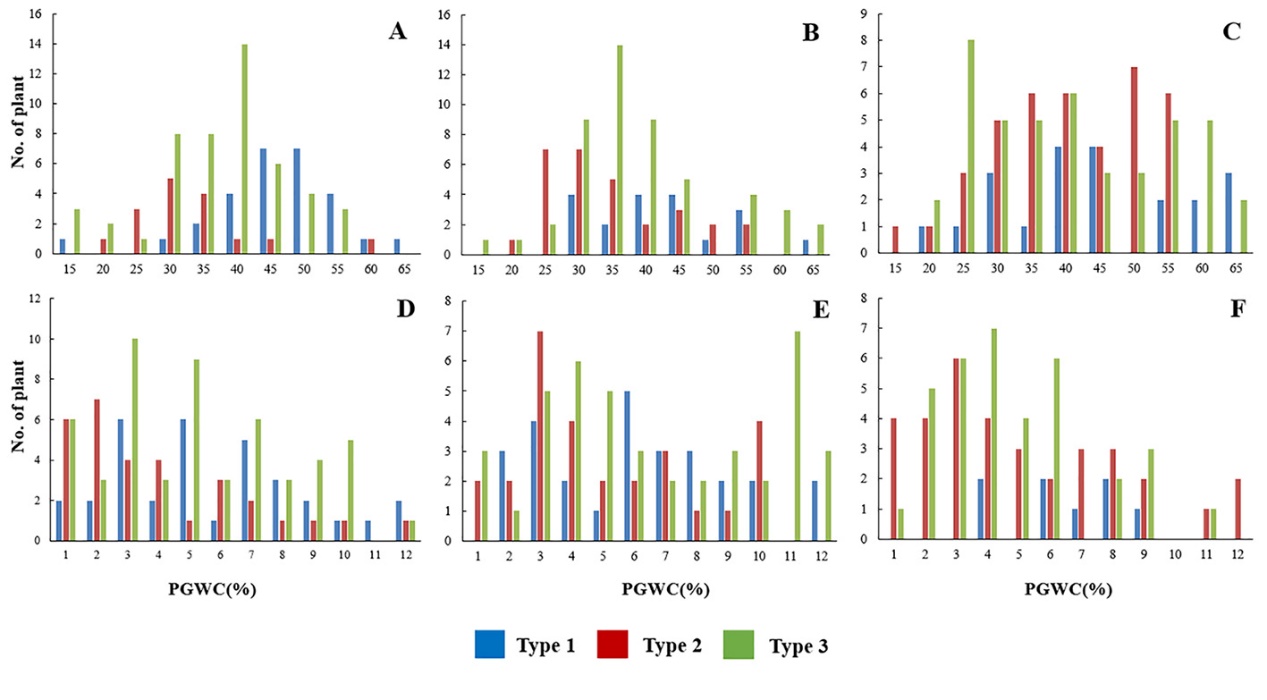
**
